# Different Roles of Dendritic Cells for Chronic Rhinosinusitis Treatment According to Phenotype

**DOI:** 10.3390/ijms23148032

**Published:** 2022-07-21

**Authors:** Junhu Tai, Jiwon Kwak, Munsoo Han, Tae Hoon Kim

**Affiliations:** 1 Department of Otorhinolaryngology-Head & Neck Surgery, College of Medicine, Korea University, Seoul 02841, Korea; junhu69@korea.ac.kr (J.T.); jwon1111@naver.com (J.K.); mshan35@gmail.com (M.H.); 2 Mucosal Immunology Institute, College of Medicine, Korea University, Seoul 02841, Korea

**Keywords:** chronic rhinosinusitis, dendritic cells, immunity, phenotype

## Abstract

Dendritic cells (DCs) are antigen-presenting cells derived from the bone marrow that play an important role in the association between the innate and adaptive immune responses. The onset and development of chronic rhinosinusitis (CRS) involve a serious imbalance in immune regulation and mechanical dysfunction caused by an abnormal remodeling process. Recent studies have shown that an increase in DCs in CRS and their function of shaping the nasal mucosal immune response may play an important role in the pathogenesis of CRS. In this review, we discuss DC subsets in mice and humans, as well as the function of DCs in the nasal sinus mucosa. In addition, the mechanism by which DCs can be used as targets for therapeutic intervention for CRS and potential future research directions are also discussed.

## 1. Introduction

Dendritic cells (DCs) are formed from bone marrow progenitors; they are essential antigen-presenting cells of the immune system, playing a wide range of roles in the association between the innate and adaptive immunity systems, antigen processing and presentation, and T-cell activation [1]. The intrinsic characteristics of DC subsets, the local tissue microenvironment, and the properties of antigens jointly determine the differentiation of T helper (Th) cells during homeostasis and inflammation into type 1 T helper (Th1), type 2 T helper (Th2), type 17 T helper (Th17), and T regulatory cells [2]. The characteristics and role of DCs in lower airway diseases have been discussed in detail [3]. Furthermore, numerous researchers have focused on the role of DCs in upper airway diseases [4]. Chronic rhinosinusitis (CRS), an upper airway disease, is a complex heterogeneous disease with various phenotypes [5]. Although numerous specific classification methods exist, the simplest and most easily recognized classification method is to divide it into two phenotypes: chronic rhinosinusitis without nasal polyps (CRSsNP) and chronic rhinosinusitis with nasal polyps (CRSwNP) [6]. The occurrence and development of CRS involves a serious imbalance in immune regulation, which includes not only an imbalance of the innate immune response but also an imbalance of the adaptive immune system [7]. Because DCs play a key role in the nasal mucosal immune response [8], the importance of DCs in the pathogenesis of CRS has attracted the attention of numerous researchers. Some studies have reported an increased number of DCs in the nasal mucosa of patients with CRS compared to a control group, especially in CRSwNP [9,10]. We review the DC subsets of mice and humans and focus on the function of DCs in nasal sinusitis mucosa with different phenotypes. Finally, the mechanism of DCs as therapeutic intervention targets for CRS, prospects for future research, and the potential clinical practicability are discussed.

## 2. Mouse DC Subsets

The two major subsets of DCs in mice are myeloid/conventional DC (cDC) and plasmacytoid DC (pDC); their phenotypes and functions are listed in Table 1. Two major lineages of mouse cDCs have been identified: cDC1 and cDC2 [11]. Mouse cDC1 includes most lymphoid resident cluster of differentiation (CD) 8α^+^ DCs and tissue resident and migrating CD103^+^ DCs [12]. Compared with other mouse DC subtypes, mouse cDC1 expresses high levels of clec9a, which can be used as a distinguishing marker [13]. XC chemokine receptor 1 (XCR1) is also one of the surface markers specifically expressed by mouse cDC1 [14]. XCR1 and clec9a require various transcription factors for development, such as basic leucine zipper ATF-like transcription factor 3 (BATF3) [15]. Mouse cDC1 presents extracellular antigens through major histocompatibility complex I (MHC I) molecules and shows strong antigen cross-presentation abilities [16]. Mouse cDC1 can drive activated CD8^+^ T cells to polarize into cytotoxic T lymphocytes [17] and can also shape the immune response in tumors [18]. cDC2 in mice is identified by the markers CD11b and signal-regulatory protein alpha (SIRPα) [19]. The development of mouse cDC2 depends on transcription factors, such as zinc finger E-box binding homeobox 2 (ZEB2) [20]. Mouse cDC2 is specifically used for MHC II presentation and the induction of CD4^+^ T cell responses, including Th1, Th2, and Th17 cells [21]. Compared to mouse cDC1, which mainly initiates the cellular immune response, mouse cDC2 mainly initiates the humoral immune response [22].

Mouse pDCs depend on FMS-like tyrosine kinase 3 ligand (Flt3L) levels during development, similar to those in the development of cDCs [23]. In addition, its development depends on specific transcriptional regulators such as basic helix-loop-helix transcription factor E2-2 [24]. Mouse pDC markers include CD45RA, CD317, and sialic acid-binding immunoglobulin-like lectin-H (Siglec-H) [25,26]. Furthermore, pDCs are the main producers of type I interferon (IFN-I) and play a role in generating immunity against viral infections [27]. Activated pDCs secrete large amounts of IFN-α/β when stimulated by toll-like receptor 7/9 (TLR7/9) [28]. pDCs also secrete a series of other inflammatory cytokines and chemokines; however, the efficiency of cDC is higher than that of pDC [29]. Inflammatory DC (inf-DC), also known as monocyte-derived DC (MoDC), is a type of DC derived from circulating blood monocytes under inflammatory conditions [30,31]. Their phenotypic spectrum is highly similar to that of cDC, which is characterized by the expression of different macrophage markers, such as CD14, CD16, CD64, Ly6C, F4/80, and so on [32]. It was shown that inf-DCs (MoDCs) play a key role in inflammatory control [33]. Mouse inf-DCs (MoDCs) can cross-present antigens to CD8^+^ T cells under certain conditions, usually in the inflammatory conditions [34,35].

## 3. Human DC Subsets

**Figure 1:**
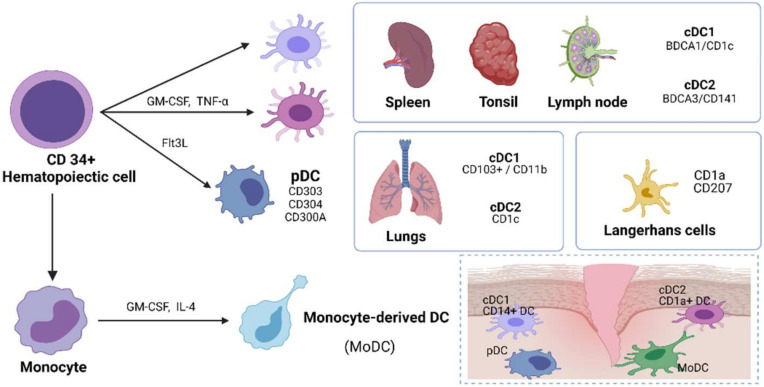
DC subsets in humans.

Human DCs are derived from hematopoietic stem cells, and their classification is developing (Figure 1). At present, DCs are divided into three main subsets: pDC, cDC1, and cDC2 [36]. pDCs have numerous surface receptors that are closely involved in the regulation of the physiological function of pDCs. Well-known surface receptors of pDCs include CD303, CD304, and CD300A [37]. Numerous studies have reported two major cDC subsets found in the spleen [38], tonsil [39], and lymph nodes [40]. cDC1 shows high expression of blood dendritic cell antigen 1 (BDCA1 or CD1c); cDC2 shows high expression of BDCA3 or CD141 [41]. In the lung, cDC1 expresses CD103 but lacks CD11b, and lung cDC2 needs to pass through CD1c [42]. Studies have shown that CD103^+^ DCs tend to induce Th1 responses, whereas CD11b^+^ DCs preferentially induce Th2 or Th17 responses [43,44]. Langerhans cells (LCs) are epidermal immune cells derived from the bone marrow that can present antigens and are in the basal and upper basal layers of the skin epidermis [45]. LCs express CD1a and the type II transmembrane molecule CD207 [46], which are continuously internalized and located in Birbeck granules [47].

Granulocyte-macrophage colony-stimulating factor (GM-CSF) is a hematopoietic growth factor that controls the differentiation of the bone marrow cell lineage [48]. The effects of GM-CSF on DCs are diverse and include DC development, antigen presentation, and survival. The differentiation of DCs also depends on Flt3L levels to a large extent [49]. Studies have shown that the injection of Flt3L in healthy human subjects increases the frequency of different DC subsets. They found that the frequency of DC subsets in pDC increased 16 times after Flt3L treatment [50]. GM-CSF and interleukin-4 (IL-4) can induce the differentiation of monocytes into DCs, which are called MoDCs [51]. In cases of infection or inflammation, monocytes are recruited to the lesion site, develop into MoDCs, and produce inflammatory cytokines to induce a unique immune response [52]. During infection, they express many receptors of cDC2, such as BDCA1, CD1a, CD11c, CD14, and CD172a [36]. Tissue-derived cDC2 typically expresses CD206, but cDC2 in blood does not express CD206 and has a low level of CD14 expression [53]. With the increasing complexity of DC subsets, people not only recognize their important role in T cell activation but also reveal their multiple functions in various diseases [54].

## 4. Phenotype Changes of DCs Based on the Infective Agents

Many studies have shown that the systemic transmission of bacterial pathogens will affect the number of DCs in the whole body and DC dysfunction. For example, Salmonella typhi infection specifically induces the death of CD8 expressing CD1 through MyD88 and TNFR1 signal transduction [55]. In the case of virus infection, the induction of IFN-I response will lead to enhanced differentiation of bone marrow cells [56]. These cells have been proved to acquire a DC phenotype during virus infection so that they can stimulate Th1 response, which is crucial for virus clearance [57]. Virus infection will change the differentiation potential and function of DC, for example, vaccinia virus infection leads to decreased myeloid progenitor cells and impaired differentiation of DCs into pDCs in vitro in a MyD88-dependent manner [58]. In addition, the production of measles virus through IFN-I is conducive to the development of cDC from pDC to cause immunosuppression [59]. In addition, in herpesvirus infection, common lymphoid progenitor cells develop into cDC due to TLR9 ligation in vitro [60]. In addition to the bacteria and viruses mentioned above, there have not been many reports on the effects of fungi on DC development. Studies have shown that *Candida albicans* yeast infection triggers the differentiation of MoDCs in a TLR2-dependent manner [61,62]. Further studies are needed to analyze the mechanism of DC differentiation and function under infection.

## 5. DC Activation

DC has pattern-recognition receptors that recognize pathogen-associated molecular patterns from viruses, bacteria, fungi, and protozoa. Among different kinds of pattern-recognition receptors, TLR is the most deeply studied [63]. Human pDCs cannot express TLR4 and do not respond to lipopolysaccharide, while CD11c^+^ DCS and MoDCs are very sensitive to lipopolysaccharide stimulation [64]. The combination of TLR and DC leads to the increased expression of costimulatory molecules and the production of immune regulatory cytokines, which have an important impact on the initiation and differentiation of T cells [65]. DC activation in response to TLR signals depends largely on the production of IFN-I and the establishment of autocrine or paracrine positive feedback loops [66]. Another major type of pattern-recognition receptor that regulates DC activation is C-type lectin [67]. DC-SIGN is one of the C-type lectins, which can respond to the signal sent by Mycobacterium tuberculosis and induce the production of IL-10 by MoDC in collaboration with lipopolysaccharide [68]. Dectin-1 is another C-type lectin. β-Glucan is a pattern-recognition receptor and can play a role in DC activation in yeast [69]. Although the role has not been determined, BDCA-2 expressed in DC has also been reported as a signal receptor [70]. The activation of DCs independent of pathogen-associated molecular patterns mainly depends on inflammatory cytokines. DCs complete the activation by recognizing endogenous host-derived molecules released by necrotizing cells [71]. Hyaluronan degradation products, fibronectin A, fibrinogen, heat shock proteins, and β-defensins are well-known endogenous TLR ligands [72]. DC can also perceive other changes in the endogenous environment. NKG2D can recognize the common stress-induced ligands in tumor cells [73]. In addition, Siglec, a family of sialic acid recognition molecules, make DC aware through the loss or change of normally expressed markers [74].

## 6. DCs in CRSsNP

The effect of DCs on CRS has always been a focus of research because they are effective antigen-presenting cells that can initiate antigen-specific T helper cell responses [75]. CRS endotypes are usually defined according to the balance of inflammatory helper T-cell patterns, which are divided into Th2 and non-Th2 endotypes [76]. DCs play a key role in Th2 or non-Th2 biased immune response polarization [77], and some studies have shown that DCs in the mucosa of patients with CRS increase, which is evidence that DCs play an important role in the occurrence and development of CRS [78].

**Figure 2:**
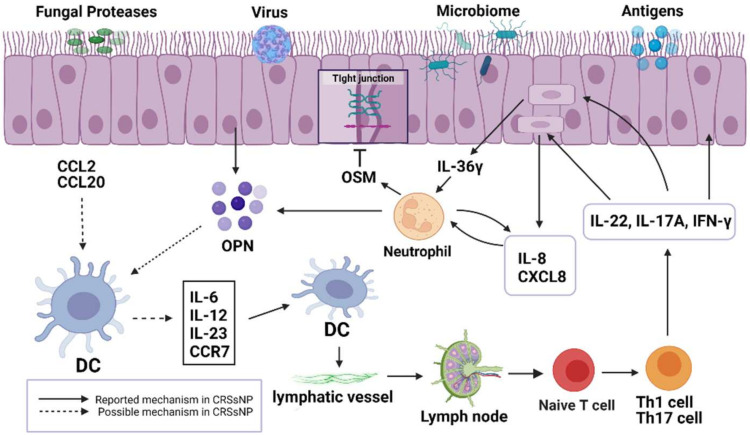
Roles of DCs in CRSsNP.

Upregulation of DCs in tissues is mainly achieved by recruiting immature DCs or monocytes differentiated into DCs in situ; furthermore, DCs can express numerous chemokine receptors [79]. Under allergen stimulation, respiratory epithelial cells can induce the production of chemokine ligand 20 (CCL20) and recruit immature DCs (Figure 2). Similarly, after allergens stimulate bronchi, high levels of CCL2, which is also a DC chemokine, can be detected in the bronchoalveolar lavage fluid [80]. An increase in CCL2 and CCL20 levels was detected in the nasal mucosa, which may be involved in the recruitment of DCs in CRS [81]. The expression level of osteopontin (OPN) in CRS is increased, which is related to DCs promoting the Th1/Th17 response [82]. OPN promotes Th1 and Th17 cell differentiation by inducing the production of IL-6, IL-12, and IL-23 in intestinal cells [83]. This process may also occur in CRS. After antigen capture and activation, DCs upregulate the expression of the C-C chemokine receptor type 7 (CCR7) on their surface and move to the lymph nodes through lymphatic vessels. Subsequently, DCs activate naïve T cells into Th1/Th17 cells and secrete IFN-γ, IL-17A, and IL-22 [84]. IL-17A can promote the expression of IL-36γ into epithelial cells [85]. IL-36γ acts on neutrophils and further aggravates neutrophil inflammation by inducing neutrophils to produce IL-8 and C-X-C motif chemokine ligand 8 (CXCL8) [86]. IL-22 directly induces epithelial cells to produce IL-8/CXCL8 [87]. Neutrophils can also produce oncostatin M (OSM) and OPN [88,89]. OSM can reduce the expression of tight junction proteins in epithelial cells and can lead to the destruction of nasal epithelial barrier function [90].

## 7. DCs in CRSwNP

Unlike CRSsNP, which shows mixed Th1 and Th2 immunophenotypes, CRSwNP is mainly associated with the type-2 immune response [91]. Th2 cells are significantly elevated in nasal polyps (NPs) of Belgian patients with CRSwNP [92]. Another study reported that increased Th2 cells were found in the NPs of Chinese patients with CRSwNP [82]. These results indicate that Th2 cells are significantly associated with CRSwNP. As mentioned earlier, DCs play a key role in distorting the Th response; therefore, the specific role of DCs in the pathogenesis of CRSwNP is important and must be the focus of research.

**Figure 3:**
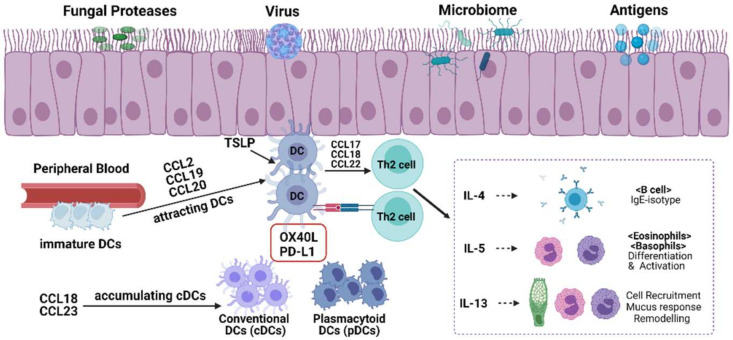
Roles of DCs in CRSwNP.

As suspected, the frequency of each subgroup of DCs increases in the NPs of patients with CRSwNP. Several studies have reported that compared with those in healthy nasal mucosa, the counts of pDCs, cDC1, and cDC2 in NPs were significantly increased [79,93]. Although several studies have shown that the number of pDCs in CRSwNP is increased, some studies have found that pDCs are downregulated in CRSwNP with asthma and that the number of pDCs in IL-5^+^IFN-γ^−^ NPS are also significantly decreased [9]. The relationship between pDCs and the CRSwNP phenotype requires further study. Some research has been conducted on the mechanism by which cDC1 participates in Th2 polarization. The researchers evaluated DC subpopulations of NPs from patients with eosinophilic CRSwNP and non-eosinophilic CRSwNP and found that cDC1 isolated from non-eosinophilic NPs could bias naive T cells to Th1 and Th17 phenotypes, but only DCs from eosinophilic NPs could bias naive T cells to Th2 phenotypes [82]. Furthermore, they found that the frequency of cDC1 expressing OX40 ligand (OX40L) or programmed death ligand-1 (PD-L1) significantly increased in eosinophilic NPs, suggesting Th2 bias caused by cDC1 (Figure 3). Blocking OX40L or PD-L1 has also been shown to inhibit Th2 bias [82]. Although cDC2 is only a small part of the cDCs in NPs compared with cDC1 in NPs, some researchers have reported that cDC2 significantly increased and strongly induced Th2 polarization in atopic asthma [94], suggesting that the increase in cDC2 in CRSwNP may be related to Th2 polarization. However, its specific mechanism remains unclear, and further in-depth studies are required. MoDCs can also present antigens and trigger the same immune response as conventional DCs [95]. Circulating MoDCs in CRSwNP are significantly increased and related to Th2 cells in the blood [75].

Chemokines are key factors that determine the recruitment of DCs to local inflammatory sites [96]. Nasal epithelial cells produce CCL20 and thymic stromal lymphopoietin (TSLP) after stimulation by fungi, viruses, microbiomes, and other antigens [84]. CCL20 can attract immature DCs from the peripheral blood vessels to the nasal sinus mucosa; in addition, CCL2 [97] and CCL19 [98] have similar functions. CCL19 can also promote contact between DCs and T cells, thereby promoting antigen presentation [99]. DC chemokines, such as CCL18 [100] and CCL23 [101], were also elevated in CRSwNP. Both CCL18 and CCL23 attract immature cDCs and play an important role in cDC1 accumulation in CRSwNP [100,101]. Another study found that CCL18 recruits Th2 cells through CCR8 [102]. However, research on cDC2-related chemokines is lacking. TSLP-stimulated DCs induce naive CD4^+^ T cells to differentiate into Th2 cells [103]. TSLP and OX40L are highly expressed in the DCs of NPs from patients with CRSwNP, and OX40L expression is associated with TSLP expression [104]. Moreover, after TSLP stimulation, DCs secrete large numbers of CCL17 and CCL22, which play a role similar to that of CCL18, both participating in the recruitment of Th2 cells [105]. Th2 cells then produce inflammatory factors such as IL4, Il5, and IL13, which participate in the inflammatory response of various cells [106]. Another study suggested that there may be an association between biofilm formation and DCs in CRSwNP. The long-term existence of various bacteria in biofilms can induce DCs to produce various cytokines involved in the mucosal immune response and aggravate the severity of CRSwNP [107].

## 8. Future Prospect of DCs as a Potential Therapeutic Target for CRS

Because DCs play a key role in nasal mucosal immune polarization, they can be considered potential therapeutic targets for CRS. At present, the therapeutic methods for CRS, such as biological agents and glucocorticoids [108], only partly regulate the function of DCs, and there is a lack of specific targeted DC pathway therapy for CRS. However, several drugs targeting DC pathways have shown promising prospects for CRS treatment. For example, researchers suggest that vitamin D3 (VD3) has the potential to act as an immune modulator. In a retrospective study, vitamin D3 levels were negatively correlated with CD209 positive NP-derived cells [109]. Some studies have also shown that an increase in VD3 downregulates the expression of costimulatory molecules and IL-10 secreted by DC [110], and researchers have shown that patients with CRS have VD3 deficiency [111]. Although a VD3 supplementary trial for patients with CRS did not achieve the expected results, the prospect of VD3 as a potential therapeutic target cannot be denied, and more studies are required to further confirm its efficacy [111]. CCX354-C [112] and BMS-817399 [113] are antagonists of CCR1 and have entered clinical trials for the treatment of rheumatoid arthritis. As antagonists of CCR1, these drugs can be considered as therapeutic agents for CRS for further research.

GM-CSF is involved in the pathogenesis of several inflammatory diseases. Some researchers have identified a new pathway for the development of inflammatory arthritis related to GM-CSF and CCL17 [114]. Their research on gene-deficient mice showed that this pathway related to CCL17 plays an important role in the development of the disease; therefore, CCL17 can be considered a potential therapeutic target for the treatment of osteoarthritis [114]. Another study proved that the expression of CCL17 during the inflammatory reaction of the peritoneum and lung was mainly regulated by GM-CSF and emphasized the role of CCL17 in driving the pain and disease progression of arthritis [115]. An experiment on the histamine 4 receptor antagonist-JNJ7777120 was conducted in patients with atopic dermatitis and a healthy control group [116]. The results showed that the production of CCL17 and CCL22 in the LC of patients in the experimental group was significantly higher than that in the control group, and the antagonist significantly inhibited the production of CCL17 and CCL22 in the LC. The experiments showed that this antagonist was feasible for the treatment of atopic dermatitis. In CRS, an inflammatory disease, CCL17 and CCL20 actively participate in the recruitment of Th2 cells. Therefore, we can conduct relevant experiments on CRS to determine whether antagonizing CCL17 can be used as a treatment option for CRS. As mentioned above, some experiments have shown that blocking OX40L or PD-L1 in CRS can inhibit the Th2 polarization function of DC [82], which is a mechanism for the DC-T cell signaling pathway. However, there is little research in this area, and more research is required to explore the specific mechanism.

## 9. Conclusions

This paper describes each subgroup of DCs in detail, summarizes the role of DCs in CRS from many aspects, and analyzes the possible therapeutic targets of DCs. In conclusion, DCs play an important role in the pathogenesis of CRS, especially in the formation of the Th reaction in the nasal sinus mucosa. In-depth research on DCs is required to develop novel therapeutic strategies targeting DC-related pathways. 

## Figures and Tables

**Table 1:** Mouse DC subsets with different phenotypes and functions.

	Phenotypes	Functions
cDC1	CD8α CD103 Clec9A XCR1	MHC I cross-presentation. Cellular immune response.
cDC2	CD11b SIRPα	MHC II presentation. Humoral immune response.
pDC	CD45RA CD317 Siglec-H	Secretion of IFN-α/β. Antiviral immune response.
Inf-DC (MoDC)	CD14 CD16 CD64 Ly6C F4/80	MHC I cross-presentation. MHC II presentation. Inflammatory control.

## Data Availability

There is no supporting data.

## Abbreviations

DCs	dendritic cells
Th	T helper
Th1	type 1 T helper
Th2	type 2 T helper
Th17	type 17 T helper
CRS	interleukin
CRSsNP	CRS without NPs
CRSwNP	CRS with NPs
XCR	XC chemokine receptor
BATF	basic leucine zipper ATF-like transcription factor
MHC	major histocompatibility complex
SIRPα	signal-regulatory protein alpha
ZEB	zinc finger E box binding homeobox
Siglec-H	sialic acid-binding immunoglobulin-like lectin-H
IFN	interferon
TLR	toll-like receptor
inf-DC	inflammatory DC
MoDC	monocyte-derived DC
pDC	plasmacytoid DC
cDC	conventional DC
BDCA	blood dendritic cell antigen
LC	langerhans cell
GM-CSF	granulocyte-macrophage colony stimulating factor
Flt3L	FMS-like tyrosine kinase 3 ligand
IL	interleukin
CCL20	chemokine ligand 20
OPN	osteopontin
CCR7	C-C chemokine receptor type 7
CXCL8	C-X-C Motif Chemokine Ligand 8
OSM	oncostatin M
NPs	nasal polyps
OX40L	OX40 ligand
PD-L1	programmed death ligand-1
TSLP	thymic stromal lymphopoietin
VD3	vitamin D3
